# T Cell Receptor Beta-Chain Profiling of Tumor Tissue, Peripheral Blood and Regional Lymph Nodes From Patients With Papillary Thyroid Carcinoma

**DOI:** 10.3389/fimmu.2021.595355

**Published:** 2021-02-18

**Authors:** Yizeng Wang, Yuanchao Liu, Li Chen, Zuoyu Chen, Xiaoning Wang, Ruoyu Jiang, Ke Zhao, Xianghui He

**Affiliations:** Department of General Surgery, Tianjin Medical University General Hospital, Tianjin Medical University, Tianjin, China

**Keywords:** papillary thyroid carcinoma, high-throughput sequencing, TCR repertoire, T cell receptor, high expansion clones

## Abstract

**Objective:** To study the characteristics of the T cell receptor (TCR) repertoire in cancer tissue, peripheral blood and regional lymph nodes (LNs) from patients with papillary thyroid carcinoma (PTC).

**Methods:** PTC tissue, peripheral blood mononuclear cells (PBMCs) and regional LNs of six patients with papillary thyroid carcinoma were harvested. T cell receptor beta-chain (TCRβ) profiling was performed though high-throughput sequencing (HTS), and IMonitor, MiXCR and VDJtools were used to analyze the characteristics of the TCR repertoire.

**Results:** The results of IMonitor and those of MiXCR and VDJtools were very similar. The unique CDR3 of TCRβ from LNs was higher than that of PBMCs, and the CDR3 of TCRβ from LNs was higher than that of PTC tissue. Shannon's diversity index, D50, inverse Simpson index_mean and normalized Shannon's diversity index_mean of CDR3 from LNs were higher than those of PTCs and PBMCs. The HEC (high expansion clones) rate of CDR3 sequences at the amino acid level in PTC tissue was higher than that of PBMCs, which was higher than that of LNs. The V-J HEC rate of CDR3 was highest in PTC tissue, followed by PBMCs and LNs.

**Conclusion:** TCR CDR3 profiling showed differences among and within the PBMCs, PTC tissues and regional LNs of PTC, including unique CDR3, CDR3 HEC at the amino acid level, CDR3 V-J HEC at the amino acid level, Shannon's diversity index and D50. The TCRβ repertoire of PTC tissue, peripheral blood and regional LNs of PTC provide a reference for further study of immunity mechanisms against PTC.

## Introduction

As an important part of the adaptive immune system, T cells can recognize potential pathogen-derived or abnormal peptides or epitopes. These peptides or epitopes are presented by the major histocompatibility complex (MHC) molecules on the cell surface of nucleated host cells or antigen-presenting cells and are recognized by T cell receptors (TCRs) on T cells to induce immune responses (1). The cellular immune response to tumor cells depends largely on TCRs and peptide-MHC complex (pMHC). TCRs are heterodimers composed of an α chain and a β chain (encoded by the TRA and TRB genes, respectively) or a γ chain and a δ chain (encoded by the TRG and TRD genes, respectively). The former is the most common, accounting for ~95% of TCRs. During T cell maturation, there is random recombination of gene segments within the variable (V), diversity (D) and joining (J) regions of the TCR gene (VDJ recombination) to generate a large number of TCRs, which can identify many specific antigens. The recombination in TCRs also produces a high degree of diversity, which is present in the V-J gene segment on the TCRα chain and in the V-D-J gene segment on the TCRβ chain. The V region of each chain of a TCR includes three hypervariable complementary-determining (CDR) regions, namely, CDR1, CDR2, and CDR3, of which CDR3 has the highest variability and is the most important region for specific recognition of an antigen (2). The TCR diversity of adults can theoretically be as high as 10^18^ (3). Therefore, the TCR repertoire is difficult to study and analyze.

The development of next-generation sequencing technology has allowed detailed studies of the immune system. In recent years, some sequencing platforms and analytical software have been developed to assess the TCR repertoire, enabling the discovery and further study of major TCR clones in different tissues and different statuses and providing new ideas for the study of T cell responses in the immune microenvironment in different diseases (4, 5).

In the past 30 years, the incidence of thyroid carcinoma, especially papillary thyroid carcinoma (PTC), has increased worldwide (6). The prognosis of PTC is good because its 20-year survival rate can exceed 90%. The underlying immune mechanism of this less aggressive biological behavior should be further studied. Targeted immunotherapy, such as PD-1 inhibitors for various malignant tumors, is the latest and most effective treatment, but some of them can induce autoimmune thyroid disease (7). The study of the immune repertoire of thyroid carcinoma will help elucidate the possible immune mechanisms mentioned above.

There are few studies about the immune repertoire of thyroid carcinoma. Lu et al. studied the relationship between neoantigens and tumor infiltrating lymphocytes (TILs) of seven noncontiguous cancer foci from a multifocal PTC patient and found that the number of nonsynonymous mutations was positively correlated with the oligoclonal TCRβ repertoire. These researchers suggested that fewer nonsynonymous somatic mutations can lead to clonal expansion of TILs, and tumors with similar mutation profiles have higher overlap of the TCRβ repertoire. Biopsies of multiple loci are required to fully describe the immune response of a multifocal tumor (8). Sun et al. performed immune repertoire sequencing on cancer tissues and adjacent tissues of five PTC patients. The length of the immunoglobulin heavy chain (IGH) CDR3 differed between the two groups. IGHV3-11/IGHJ6, TRBV2/TRBJ1-2 and TRBV2/TRBJ1-1 may be progressive markers of PTC. The Shannon index of cancer tissues is relatively low, while the number of highly amplified clones is relatively high (9). However, no studies have been published on the immune repertoire of regional lymph nodes (LNs) of the thyroid and peripheral blood of PTC patients. In this study, TCRβ CDR3 sequencing was performed on the peripheral blood mononuclear cells (PBMCs), PTC tissue and regional LNs of six PTC patients, and preliminary analysis was performed to provide a reference for further immune-related research.

## Materials and Methods

### Patients and Samples

In this study, six patients were enrolled in the General Surgery Department of Tianjin Medical University General Hospital (Tianjin, China) and underwent thyroidectomy plus central lymph node dissection. These six patients were diagnosed with PTC by analysis of postoperative paraffin sections. None of them had a history of thyroid carcinoma surgery. The specimens of PTC tissues and LNs were split such that half were used for pathological testing, while the other half were used for T cell extraction and further sequencing. Peripheral blood was collected for PBMC preparation and further experiments. This study was approved by the Institutional Review Board of Tianjin Medical University General Hospital. Informed consent was preoperatively provided by all patients.

### Cell Isolation and DNA Extraction

PBMC isolation from peripheral blood and lymphocyte isolation from LNs and cancer tissue were performed using density gradient centrifugation technology following the manufacturer's instructions for Human Lymphocyte Separation Medium LTS1077 and LTS1077Z (Tianjin HaoYang Biological Manufacture Co., Tianjin, China), respectively. DNA was extracted from PBMCs and lymphocytes using a TIANamp Genomic DNA Kit (Tiangen Biotech (Beijing) Co., Beijing, China) extraction kits according to the manufacturer's instructions.

### Library Construction and Sequencing

In this study, we used PCR to construct the libraries PCR1 and PCR2 inclusively and semiquantitatively. During the first round of PCR1, only 10 cycles were used to amplify CDR3 fragments using specific primers against each V and J gene. In the second round, PCR was performed using universal primers.

#### PCR1

A total of 600 ng of DNA (used as templates) was amplified after 25 μL of 2×Qiagen Multiplex PCR Master Mix, 5 μL of 5×Q solution, 1 μL of forward primer set pool, and 1 μL of reverse primer set pool were added to form a reaction system by using a Multiplex PCR Kit (Qiagen, Germany). Then, PCR was performed at 1 cycle of 95°C for 15 min, 10 cycles of denaturation at 94°C for 30 s, and 15 cycles of both annealing at 60°C for 90 s and extension for 30 s at 72°C. After a final extension for 5 min at 72°C, the system was cooled to 4°C. The target fragment of multiplex PCR products was purified on magnetic beads (Agencourt No. A63882, Beckman, Beverly, MA, USA).

#### PCR2

All samples of the PCR1 product were used as templates for a second step of amplification following the addition of 2 μL of communal primers, 25 μL of Phusion master mix prepared using the Phusion^®^ High-Fidelity PCR Kit (New England Biolabs, America), and nuclease-free water to reach a total volume of 50 μL. The reactions were then transferred to a thermal cycler that carried out the following program: one cycle of 98°C for 1 min; 25 cycles of denaturation at 98°C for 20 s, annealing at 65°C for 30 s and extension at 72°C for 30 s; and a final extension at 72°C for 5 min. The samples were then held at 4°C. Size selection was performed by agar gel electrophoresis (400 mA/100 V, 2 h), and target fragments between 200 and 350 bps were retrieved and purified by a QIAquick Gel Purification Kit (Qiagen, Germany). After gel purification, the PCR product was subjected to high-throughput sequencing (HTS) using the Illumina NovaSeq 6000 platform.

### Data Analysis

The TCR repertoire analyzing pipeline IMonitor was used to analyze sequencing data, and the default parameters of IMonitor were used for analysis. IMonitor developed by BGI to analyze the TCR repertoire and B cell receptor repertoire of next-generation sequencing technology. IMonitor analyzes and processes data in four steps: basic data processing, V (D) J assignment, structural analysis and statistical visualization. Adaptor sequence and low-quality bases (base quality < 10) were detected and discarded and the PE reads were merged for basic data processing. The V (D) J assignment program takes the BLAST alignment results as input, realigns the sequence to reference from IMGT database for both the non-CDR3 region and the CDR3 region, calculating the alignment score and identity, mismatch number, and alignment length, and then selects the maximal score as the best hit. In structural analysis process, PCR and sequencing error correction were done, then DNA was translated to protein and CDR3 region identified, and the effective data was obtained after filtration. The basic statistics of IMonitor include CDR3 frequency distribution, V-J paring, V/J usage, CDR3 length distribution, CDR3 segmental frequency statistics, etc. And figures were plotted for statistical visualization. One of the important features of IMonitor software is its realignment process. In this process, the CDR3 region uses the M-mismatch expansion model for local comparison, while the non-CDR3 region is used for overall comparison. In addition, this software can correct PCR and sequencing errors and minimize MPCR bias. Moreover, the results of the IMonitor are displayed in intuitive graphs (10).

MiXCR is a universal framework that processes TCR repertoire data from raw sequences to quantitated clonotypes. MiXCR is a very simple, yet fast and accurate tool for T- and B- cell receptor repertoire extraction (11). VDJtools is a software that can analyze output of most commonly used TCR repertoire processing tools. The immune repertoire post-analysis results by VDJtools can be subdivided into several analysis modules, including basic analysis, diversity estimation and repertoire overlap analysis (12). MiXCR v3.0.13 and VDJtools 1.2.1 were also used to analyze the sequencing data (the default parameters were used). The results were generally written in parentheses after the IMonitor results.

### Statistical Analysis

R 3.6.2 and GraphPad Prism 8 were used for statistical analysis and drawing. One-way ANOVA was used to compare differences among three groups, and Tukey's multiple comparisons test was used to compare differences between two groups. *p* < 0.05 was considered statistically significant. In the illustrations in this article, * represents a *p*-value range of 0.01–0.05, ** represents a *p*-value range of 0.001–0.01, *** represents a *p*-value range of 0.0001–0.001, and **** represents a *p*-value range < 0.0001.

## Results

### The Basic Characteristics of the TCR Repertoire by IMonitor and by MiXCR and VDJtools

**Figure 1:**
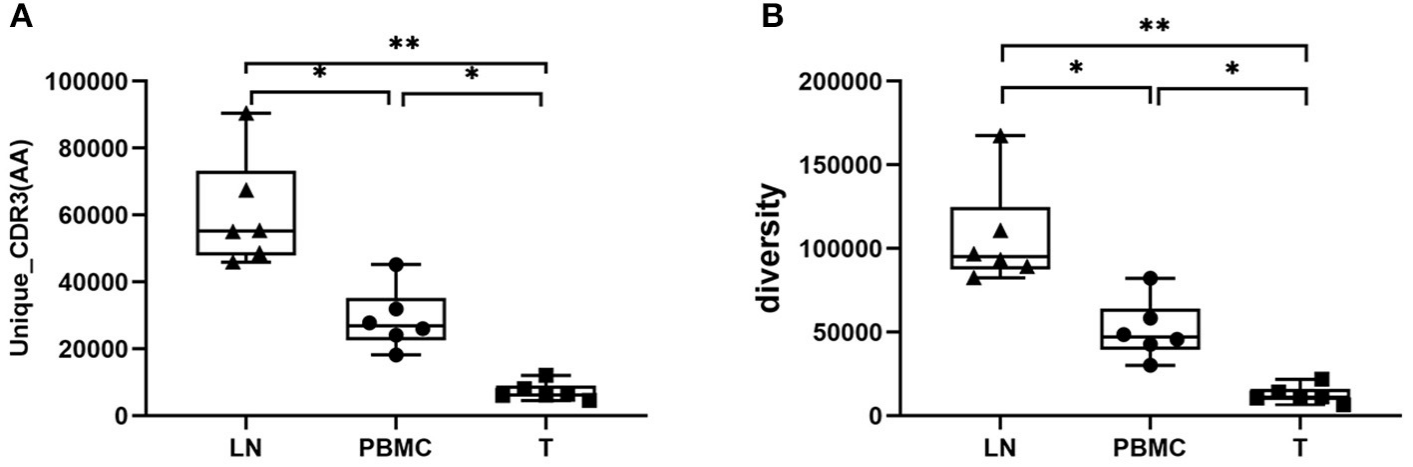
Comparison of the mean number of CDR3 clonotypes (unique CDR3 or “diversity”) of PBMCs, LNs and PTC tissues (T). One-way ANOVA and Tukey's multiple comparisons test were used. Significant differences were found among the three groups: **(A)** shows the result of unique CDR3 analyzed followed IMonitor (*p* = 0.0002), **(B)** shows the result of “diversity” analyzed followed MiXCR and VDJtools (*p* = 0.0003). The mean value of unique (“diversity”) CDR3s of LNs [60,492 (106,675)] was higher than that of PBMCs [28,896 (51,296)], and both were higher than that of PTC tissues (T) [7,277 (12,566)]. * represents a p value range of 0.01–0.05, ** represents a p value range of 0.001–0.01.

The basic characteristics of the TCR repertoire by IMonitor and by MiXCR and VDJtools are presented in Table 1 and Table 2, respectively. The average raw reads of PBMCs, LNs, and PTC tissues by IMonitor were 8,885,766, 9,024,276, and 8,927,650, respectively, and there was no significant difference among the three groups (one-way ANOVA, *p* = 0.570), indicating that the sequencing depth was consistent. The average total CDR3 values of PBMCs, LNs, and PTC tissues by IMonitor were 6,515,476, 6,453,444, and 5,272,894, respectively. There was no significant difference among the three groups (one-way ANOVA, *p* = 0.157), indicating that the removed low-quality and mixed sequences were consistent and that the samples had sequencing consistency. The average reads of PBMCs, LNs, and PTC tissues by MiXCR and VDJtools were 8,116,006, 8,273,136, and 6,520,701, respectively, and there was no significant difference among the three groups (one-way ANOVA, *p* = 0.7766). The unique CDR3 (diversity) mean values at the amino acid level of the PBMCs, LNs, and PTC tissues by IMonitor (MiXCR and VDJtools) were 28,896 (51,296), 60,492 (106,675), and 7,277 (12,566), respectively, and significant differences were found among the three groups [one-way ANOVA and Tukey's multiple comparisons test, *p* = 0.0002 (0.0003), Figures 1A,B]. That is, the number of unique CDR3s of the regional LNs was higher than that in the PBMCs, which was higher than that in the PTC tissues. The average lengths of the CDR3 amino acid sequences of the PBMCs, LNs, and PTC tissues by IMonitor were 12.649, 12.492, and 12.623 amino acids, respectively, and there was no significant difference among these groups (one-way ANOVA, *p* = 0.297). The average lengths of the CDR3 nucleotide sequence of the PBMCs, LNs, and PTC tissues were 45.98, 45.70, and 45.61 amino acids, respectively, and there was no significant difference among these groups (one-way ANOVA, *p* = 0.6587).

**Table 1:** The basic characteristic of TCR repertoire by IMonitor.

**Sample**	**Raw reads**	**Total CDR3 (AA)**	**Unique CDR3 (AA)**	**Shannon**	**D50**	**Average length of CDR3 (AA)**	**HEC (0.1%) rate^a^**
TGS2019001p	8,327,147	6,120,140	27,822	12.544	8.252	12.732	0.00158
TGS2019001n	8,920,715	6,656,041	55,386	15.063	18.470	12.364	0.00009
TGS2019002p	7,642,562	6,036,915	26,072	9.972	1.266	12.48	0.0015
TGS2019002n	7,391,292	5,539,104	45,945	14.635	16.420	12.157	0.00017
TGS2019001t	7,995,766	5,385,031	6,166	10.17	4.590	12.419	0.02108
TGS2019002t	8,056,050	5,491,435	8,089	10.074	2.547	12.805	0.01793
TGS2019003p	7,774,449	5,910,036	24,086	8.714	0.0250	12.555	0.00091
TGS2019003n	8,058,827	5,715,046	48,599	14.92	20.659	12.725	0.00019
TGS2019003t	12,452,473	7,372,585	12,128	11.414	4.502	12.734	0.00973
TGS2019004p	10,292,611	7,501,180	31,970	13.572	13.388	12.785	0.00185
TGS2019004n	11,049,016	8,237,225	90,488	15.845	20.810	12.668	0.00004
TGS2019004t	9,391,843	4,687,051	6,169	10.954	8.964	12.634	0.01572
TGS2019005p	10,053,554	7,260,337	45,192	14.154	16.383	12.562	0.00042
TGS2019005n	9,375,529	5,996,148	55,079	15.167	21.558	12.536	0.00009
TGS2019005t	8,404,630	4,084,843	4,651	10.095	6.321	12.376	0.02193
TGS2019006p	9,224,271	6,264,245	18,234	9.367	0.121	12.777	0.00258
TGS2019006n	9,350,275	6,577,101	67,454	15.438	21.724	12.502	0.00009
TGS2019006t	7,265,135	4,616,417	6,461	10.264	2.972	12.77	0.02291
Average PBMC	8,885,766	6,515,476	28,896	11.387	6.572	12.649	0.00147
Average LN	9,024,276	6,453,444	60,492	15.178	19.940	12.492	0.000112
Average T	8,927,650	5,272,894	7,277	10.495	4.983	12.623	0.0182

a *HEC rate indicated that the TCR CDR3 clones accounted for more than 0.1%*.

**Table 2:** The basic characteristics of the TCR repertoire by MiXCR and VDJtools.

**Sample**	**Reads**	**Diversity**	**Normalized shannon wiener index_mean**	**Inverse simpson index_mean**	**Mean_cdr3nt_length**	**HEC (0.1%) rate**
TGS2019001p	7,774,266	45,678	0.8410	181.8842	45.1110	0.001281
TGS2019001n	8,348,147	93,321	0.9434	26,535.5560	44.3714	0.00007670
TGS2019002p	7,056,479	48,518	0.6990	18.4504	44.6728	0.001142
TGS2019002n	6,876,207	82,588	0.9269	14,861.5995	43.3007	0.0001045
TGS2019001t	6,457,010	10,785	0.8050	275.5922	44.8601	0.02119
TGS2019002t	6,489,392	14,204	0.7681	243.7460	45.6468	0.01669
TGS2019003p	7,128,505	42,743	0.6257	20.1434	46.1349	0.0008658
TGS2019003n	7,271,651	89,262	0.9381	17,574.2973	47.2510	0.0001404
TGS2019003t	9,454,086	21,973	0.8322	719.3142	46.3142	0.009202
TGS2019004p	9,396,446	58,368	0.8932	1,746.8823	46.3678	0.001610
TGS2019004n	10,309,795	167,458	0.9492	37,261.2445	46.1579	0.00003700
TGS2019004t	5,745,140	10,516	0.8539	755.2792	45.2220	0.01543
TGS2019005p	9,024,787	82,188	0.9058	1,101.2169	46.3850	0.0003684
TGS2019005n	8,294,235	96,738	0.9472	25,102.8850	47.0410	0.00007883
TGS2019005t	5,039,652	6,546	0.8367	334.0823	44.6685	0.02516
TGS2019006p	8,315,552	30,282	0.6583	69.0650	47.2151	0.002334
TGS2019006n	8,538,782	110,681	0.9518	21,517.9941	46.1019	0.00006501
TGS2019006t	5,938,924	11,372	0.7884	371.3874	46.9438	0.02038

### Diversity

Shannon's diversity index uses information theory to reflect the diversity of communities. The higher the community diversity, the richer the species is, and the higher the evenness, the higher Shannon's diversity index is. D50 is an indicator of the level of diversity, defined as the smallest percentage of different CDR3s that make up at least half of the total CDR3s in a population or subpopulation of immune system cells. The higher the value, the higher the clone diversity is. Inverse Simpson index is the effective number of clonotypes. The higher the index, the more the clonotypes is.

**Figure 2:**
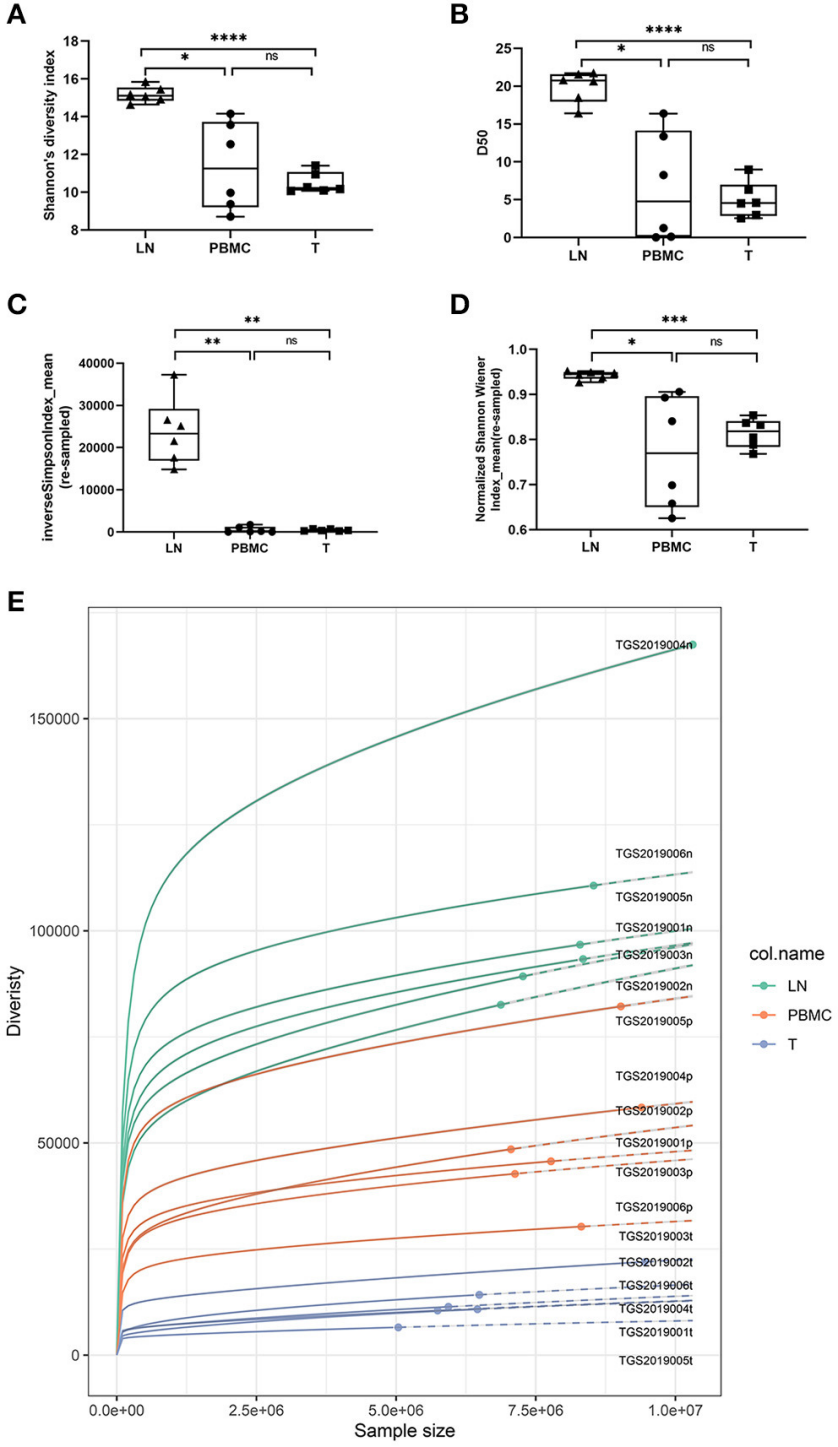
Comparison of Shannon's diversity index [**(A)**, IMonitor], D50 [**(B)**, IMonitor] The inverse Simpson index_mean [**(C)**, VDJtools] and normalized Shannon's diversity index_mean [**(D)**, VDJtools] of CDR3s among the PBMCs, LNs and PTC tissues (T). Statistical analysis methods: One-way ANOVA and Tukey's multiple comparisons test (Shannon's diversity index: *p* = 0.0059, D50: *p* = 0.0017, the inverse Simpson index_mean: *p* = 0.0006, normalized Shannon's diversity index_mean: *p* = 0.0197). * represents a p value range of 0.01–0.05, ** represents a p value range of 0.001–0.01, and **** represents a p value range < 0.0001. The median value of the LNs was significantly higher than that of the PBMCs and PTC tissues. Although the median of the PBMCs was higher than that of the PTC tissues, there was no significant difference. **(E)** is the Rarefaction plot by VDJtools. The plot indicated that sufficient observations had been made to get a reasonable estimate of diversity, and the diversity of LNs was the highest and that of the PTC tissues was the lowest.

In this study, Shannon's diversity index and D50 of the LNs, PBMCs and PTC tissues by IMonitor were significantly different (one-way ANOVA and Tukey's multiple comparisons test, *p* < 0.05, Figures 2A,B). Inverse Simpson index_mean and normalized Shannon's diversity index_mean of the LNs, PBMCs and PTC tissues by MiXCR and VDJtools were significantly different (one-way ANOVA and Tukey's multiple comparisons test, *p* < 0.05, Figures 2C,D. According to the suggestions from the author of VDJtools, the results of re-sampled data were used for between-sample comparisons). The diversity LNs was higher than that of the PTC tissues and the PBMCs, and there was no significant difference between the PBMCs and PTC tissues. The Rarefaction plot by VDJtools indicated that sufficient observations had been made to get a reasonable estimate of diversity. The diversity of LNs was the highest and that of the PTC tissues was the lowest in the plot (Figure 2E).

The inverse Simpson index_mean and normalized Shannon's diversity index_mean were similar to Shannon's diversity index and D50, of which that of the LNs was higher than that of the PTC tissues and the PBMCs, and there was no significant difference between the PBMCs and PTC tissues (Figure 2).

### CDR3 HEC at the Amino Acid Level

**Figure 3:**
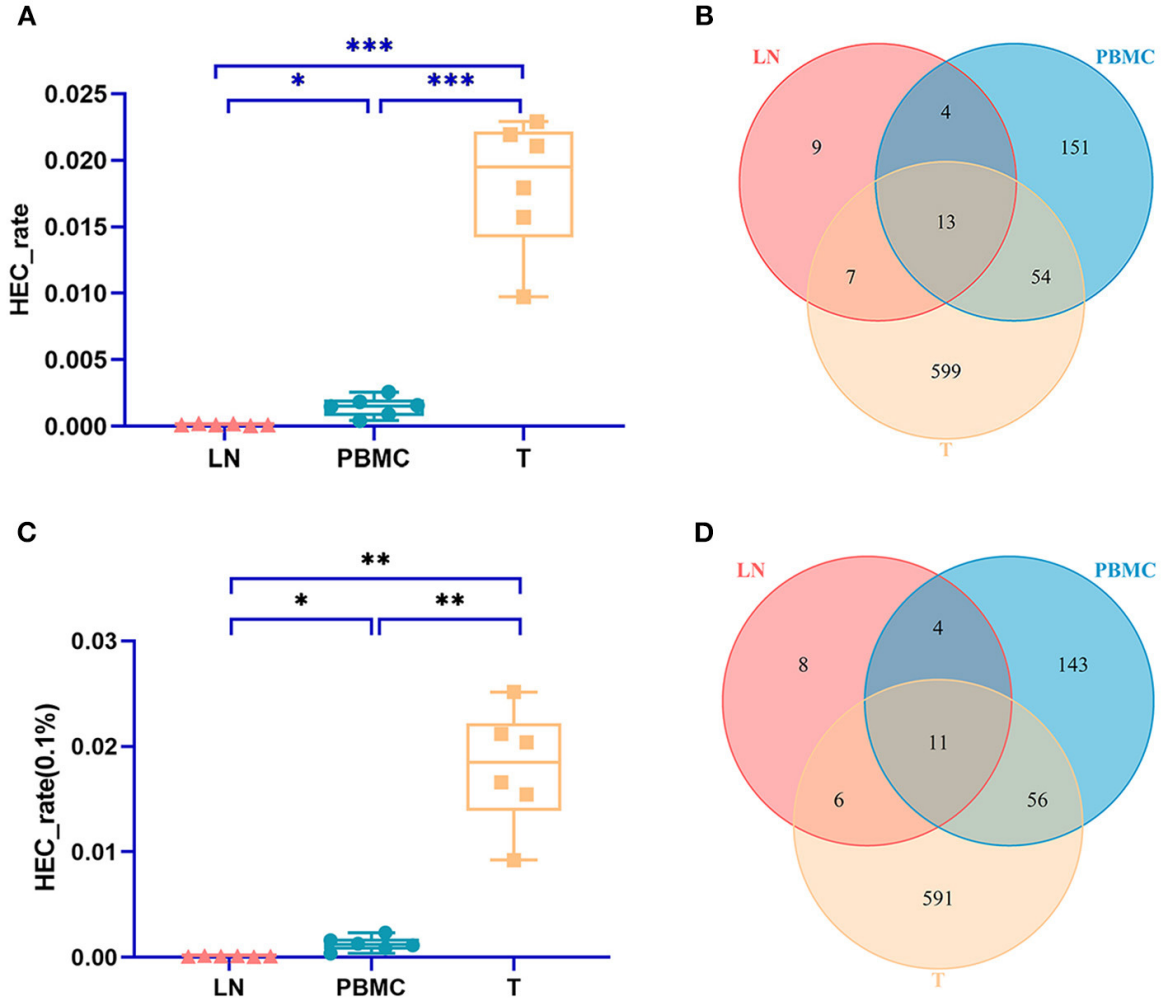
Comparison of the HEC number of CDR3 sequences at the amino acid level of the PBMCs, LNs and PTC tissues (T). **(A)** is a statistical comparison of the HEC rate among the PBMC, LN, and PTC groups by IMonitor (*p* = 0.0003) [**(C)** was that result by MiXCR and VDJtools, *p* = 0.0006]. The statistical analysis methods were one-way ANOVA and Tukey's multiple comparisons test. * represents a p value range of 0.01–0.05, ** represents a p value range of 0.001–0.01, *** represents a p value range of 0.0001–0.001. The HEC rate of CDR3 sequences at the amino acid level of the PTC tissues (T) was higher than that of the PBMCs, which was higher than that of the LNs. **(B,D)** shows the Venn diagrams of the HECs of the three groups followed IMonitor and VDJtools, respectively.

The TCR CDR3 clones accounting for more than 0.1% were defined as HECs (high expansion clones) in this study. The PBMC group, LN group, and T group had significant differences in the HEC rate by IMonitor (MiXCR and VDJtools) [one-way ANOVA and Tukey's multiple comparisons test, *p* = 0.0003 (0.006), Figures 3A,C]. There are 13 (11) CDR3 amino acid sequences shared among the three groups. There were 9 (8) unique CDR3 sequences in the LN group, 151 (143) unique CDR3 sequences in the PBMC group, and 599 (591) unique CDR3 sequences in the PTC group (Figures 3B,D). The number of CDR3 HECs in the cancer tissue sample was higher than that in the peripheral blood sample, and both were higher than that in the LN sample.

There were 33 (32) HECs in the LNs by IMonitor (MiXCR and VDJtools). Among the six patients, TGS2019003 had the most LN HECs (9 (8) HECs). No HEC was common to all patients. The CDR3 amino acid sequence ASSTYRDRVNYGYT (CASSTYRDRVNYGYTF) was shared by four patients (TGS2019003, TGS2019004, TGS2019005, TGS2019006). Its corresponding V-J rearrangement was TRBV19/TRBJ1-2; this sequence was not found in the other two patients with non-HEC CDR3 amino acid sequences. And this sequence was also found in the HECs of four patients (TGS2019003, TGS2019004, TGS2019005, TGS2019006) in the PBMCs and PTC tissues. Most LN HECs are unique to each patient. In the LN group, ASKASQGYT (CASKASQGYTF) accounted for ~0.472% (0.441%) of the sample TGS2019006n, which was the highest among the six patients. This sequence was also present in the samples TGS2019006p [1.245% (1.183%)] and TGS2019006t [0.255% (0.251%)] of this patient but not in the HECs of the other five patients in the PBMC group, PTC tissue group and LN group.

There were 222 (214) HECs in the PBMCs by IMonitor (MiXCR and VDJtools). Among the six patients, TGS2019004 had the most PBMC HECs [59 (58) HECs]. No HEC was common to all patients. Two CDR3 amino acid sequences were shared by four patients (TGS2019003, TGS2019004, TGS2019005, TGS2019006): ASSLYGPGNEQY(CASSLYGPGNEQYF), whose corresponding V-J rearrangement was TRBV27/TRBJ2-7, and ASSTYRDRVNYGYT(CASSTYRDRVNYGYTF), whose corresponding V-J rearrangement was TRBV19/TRBJ1-2. Similar to LN HECs, most PBMC HECs are unique to each patient. In the PBMCs, ASSEGTGGGETQY (CASSEGTGGGETQYF) accounted for ~27.461% (26.320%) of the sample TGS2019002p, which was the highest among the six patients, but it was not found in the HECs of the other five patients in the PBMCs, PTC tissues and LNs or in samples TGS2019002t and TGS2019002n of this patient. ASSLEGGVISNQPQH (CASSLEGGVISNQPQHF) [19.153% (18.802%)], ASSLLHEAF (CASSLLHEAFF) [14.386% (14.357%)], and ASSLRDSNTGELF (CASSLRDSNTGELFF) [12.658% (12.496%)] were detected in sample TGS2019003p; all were present in this patient's sample TGS2019003t. These three peptides were not present in sample TGS2019003n or in the HECs of the other five patients in the PBMCs, PTC tissues and LNs.

There were 673 (737) HECs in PTC tissues by IMonitor (MiXCR and VDJtools), and TGS2019006 (TGS2019002) had the most T HECs [148 (146) HECs] among the six patients. No HEC was common to all patients. Similar to LN HECs and PBMC HECs, most T HECs were unique to each patient. There were 13 CDR3 amino acid sequences shared by four patients (TGS2019003, TGS2019004, TGS2019005, TGS2019006). Among them, ASIFPRTYKAF (CASIFPRTYKAFF), ASSESGGSYYNEQ (CASSESGGSYYNEQFF), ASSIGRGNTEAF (CASSIGRGNTEAFF), ASSLFPAGGITGELF (CASSLFPAGGITGELFF), ASSYGFDEQF (CASSYGFDEQFF), ATSKTGTANYGYT (CATSKTGTANYGYTF) did not exist in the HECs of the LNs and PBMCs. In PTC tissues, ASSLQGDTEAF (CASSLQGDTEAFF) accounted for 5.665% (5.506%) of the sample TGS2019001t, which was the highest among the six patients. It also accounted for 0.161% (0.151%) of sample TGS2019002t and 0.110% (0.105%) in sample TGS2019001p. This sequence was not found in TGS2019002p and TGS2019002n or in the HECs of the other four patients in the PBMCs, PTC tissue and LNs. The second sequence is ASGQGSREAF (CASGQGSREAFF) [4.245% (4.119%)] in sample TGS2019005t. The third sequence is SVLTADRGDLRSMNTEAF (CSVLTADRGDLRSMNTEAFF) [3.979% (3.859%)] in TGS2019002t.

**Figure 4:**
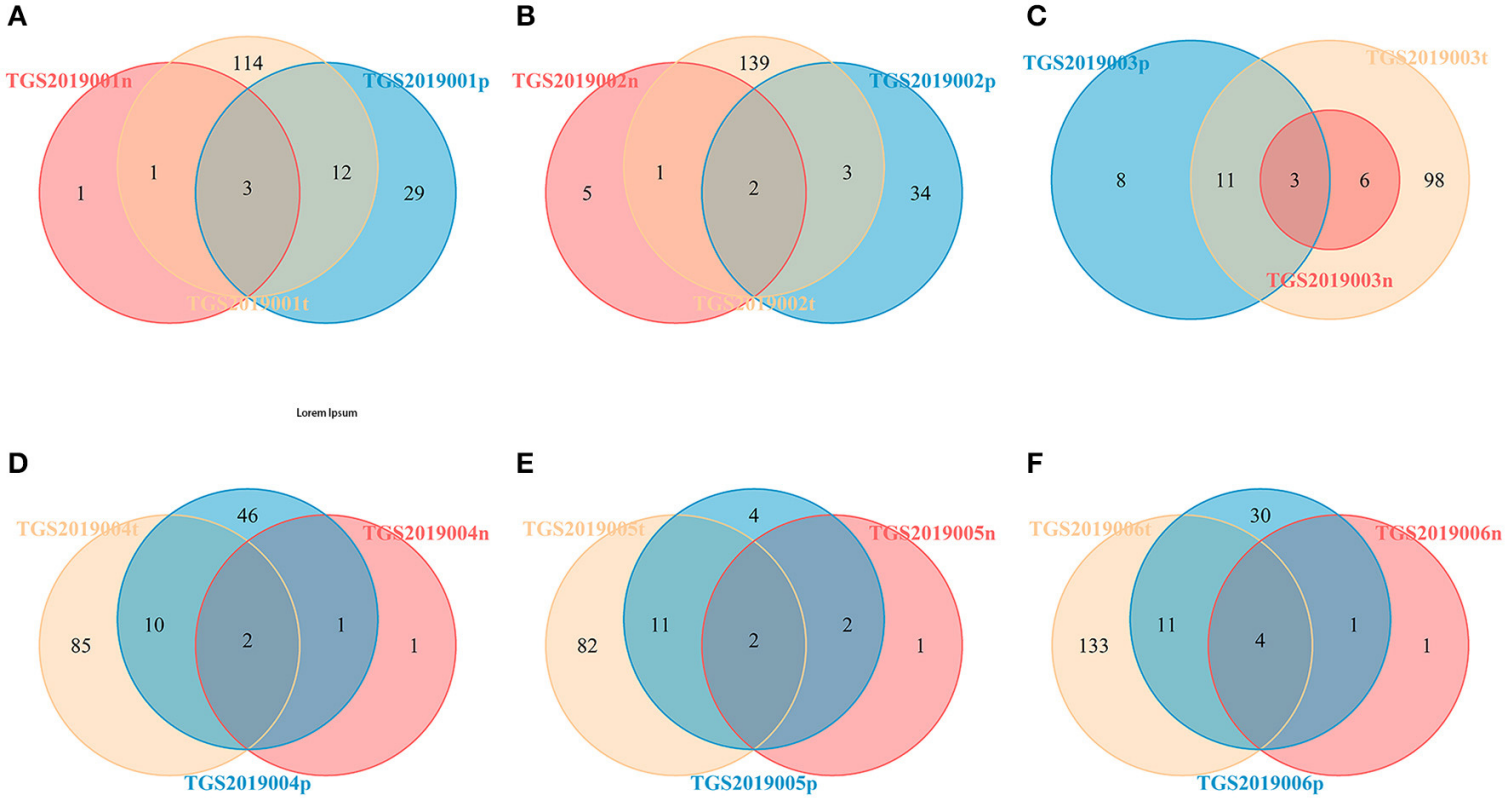
Venn diagram of the HECs of PBMCs, LNs and PTCs from six patients [TGS2019001 **(A)**, TGS2019002 **(B)**, TGS2019003 **(C)**, TGS2019004 **(D)**, TGS2019005 **(E)**, and TGS2019006 **(F)**]. “-t” indicates PTC tissues; “-n” indicates LNs; “-p” indicates PBMCs. The number of CDR3 HECs at the amino acid level shared by the PBMC, LN, and PTC samples in each patient was 2–4.

For each patient, the number of CDR3 HECs at the amino acid level shared by the PBMC, LN, and PTC samples in each patient was 2–4. The number of unique HECs in the PTC tissue was greater than that in the PBMC samples, and both values were higher than that in the LN samples (Figure 4).

### CDR3 V-J Recombination of HECs at the Amino Acid Level by IMonitor

**Figure 5:**
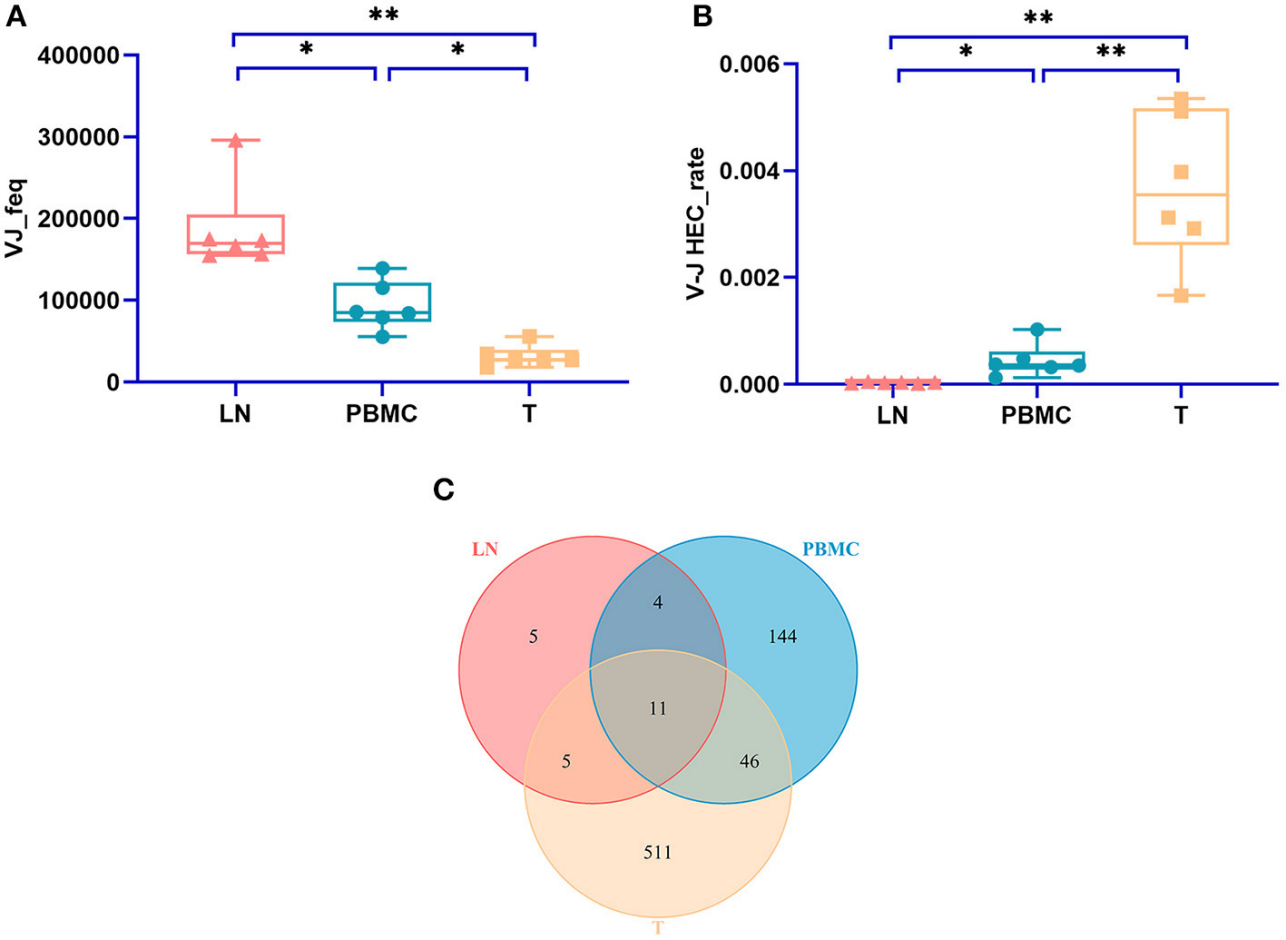
The number V-J rearrangements and V-J HEC-related statistics by IMonitor. **(A)** shows a comparison of the number of V-J recombinations of CDR3 at the amino acid level in the PBMCs, LNs, and PTC tissues (T). The differences among the three groups were significant (one-way ANOVA and Tukey's multiple comparisons test, *p* = 0.0002). **(B)** is a comparison of the V-J HEC rate among the three groups. Statistically significant differences in the V-J HEC rate were found among the three groups (one-way ANOVA and Tukey's multiple comparisons test, *p* = 0.0013). **(C)** is a Venn diagram of the number of V-J HECs in the three groups. There were 726 V-J HECs in the three groups in total. * represents a p value range of 0.01–0.05, ** represents a p value range of 0.001–0.01.

The numbers of V-J recombinations of CDR3 at the amino acid level in the PBMCs, LNs, and PTC tissues were 93,297.333, 187,014.167, and 31,787.667, respectively. The differences between these groups were significant (one-way ANOVA and Tukey's multiple comparisons test, *p* = 0.0002, Figure 5A). The number of V-J recombinations in the LNs was higher than that in the PBMCs, and the numbers of both groups were higher than that of the PTC tissues.

Similar to TCR CDR3 HECs at the amino acid level, V-J HECs were defined as the number of V-J recombinations of TCR CDR3 at the amino acid level exceeding 0.1%. There were 726 V-J HECs in PBMCs, PTC tissues and LNs, including 205 V-J HECs in PBMCs, 572 V-J HECs in PTC tissues and 25 V-J HECs in LNs. Eleven V-J HECs were shared in all three groups, 511 unique V-J HECs were in PTC tissues, 144 unique V-J HECs were in PBMCs, and five unique V-J HECs were in LNs (Figure 5C). Significant differences in the V-J HEC rate were found among the three groups (one-way ANOVA and Tukey's multiple comparisons test, *p* = 0.0013, Figure 5B). The V-J HEC rate of the PTC tissues was highest, followed by that of the PBMCs and LNs.

None of the 25 V-J HECs were shared by all samples in the LN group. TRBV19/TRBJ1-2, corresponding to ASSTYRDRVNYGYT, was present in four patients (TGS2019004, TGS2019005, TGS2019006, TGS2019003) and was also present in these four patients' M samples and PBMC samples. It accounts for 0.318% of sample TGS2019003n, which was the highest. Furthermore, TGS2019003n had the most V-J HECs (8 V-J HECs).

None of the 205 V-J HECs were shared by all samples in the PBMC group. Two V-J HECs were shared by four patients (TGS2019003, TGS2019004, TGS2019005, TGS2019006): TRBV19/TRBJ1-2 (corresponding to ASSTYRDRVNYGYT) (also present in these four patients' M samples and LN samples) and TRBV27/TRBJ2-7 (corresponding to ASSLYGPGNEQY). TGS2019006p had 57 V-J HECs, which was the highest number. The highest V-J HEC rate was 27.301%, and the corresponding V-J recombination was TRBV2/TRBJ2-5 (ASSEGTGGGETQY) in sample TGS2019002p. TRBV2/TRBJ2-5 was not found in the other five patients' V-J HECs.

**Figure 6:**
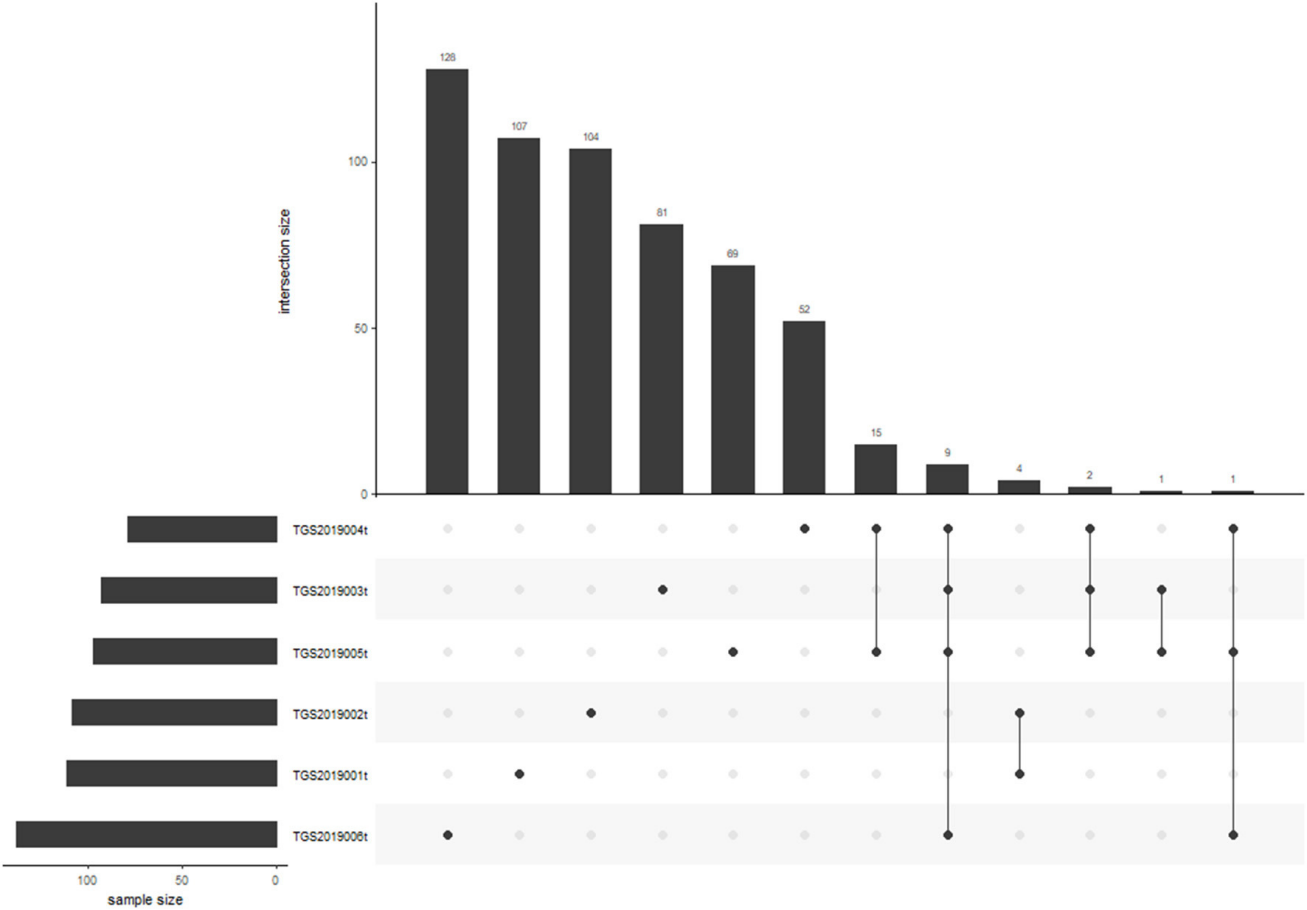
V-J HEC distribution in each sample of PTC tissue by IMonitor. No V-J HEC was shared in any of the six samples. TGS2019006t had the most V-J HECs with 138.

Nine of 572 V-J HECs were shared by four patients in the PTC tissue group. TGS2019006t had the most V-J HECs with 138 (Figure 6). Among the V-J HECs, TRBV7-9/TRBJ1-1 (ASSLQGDTEAF) had the highest percent in patient TGS2019001t and accounted for 5.451%. It was not found in the V-J HECs of the other five patients' PBMC samples and LN samples.

**Figure 7:**
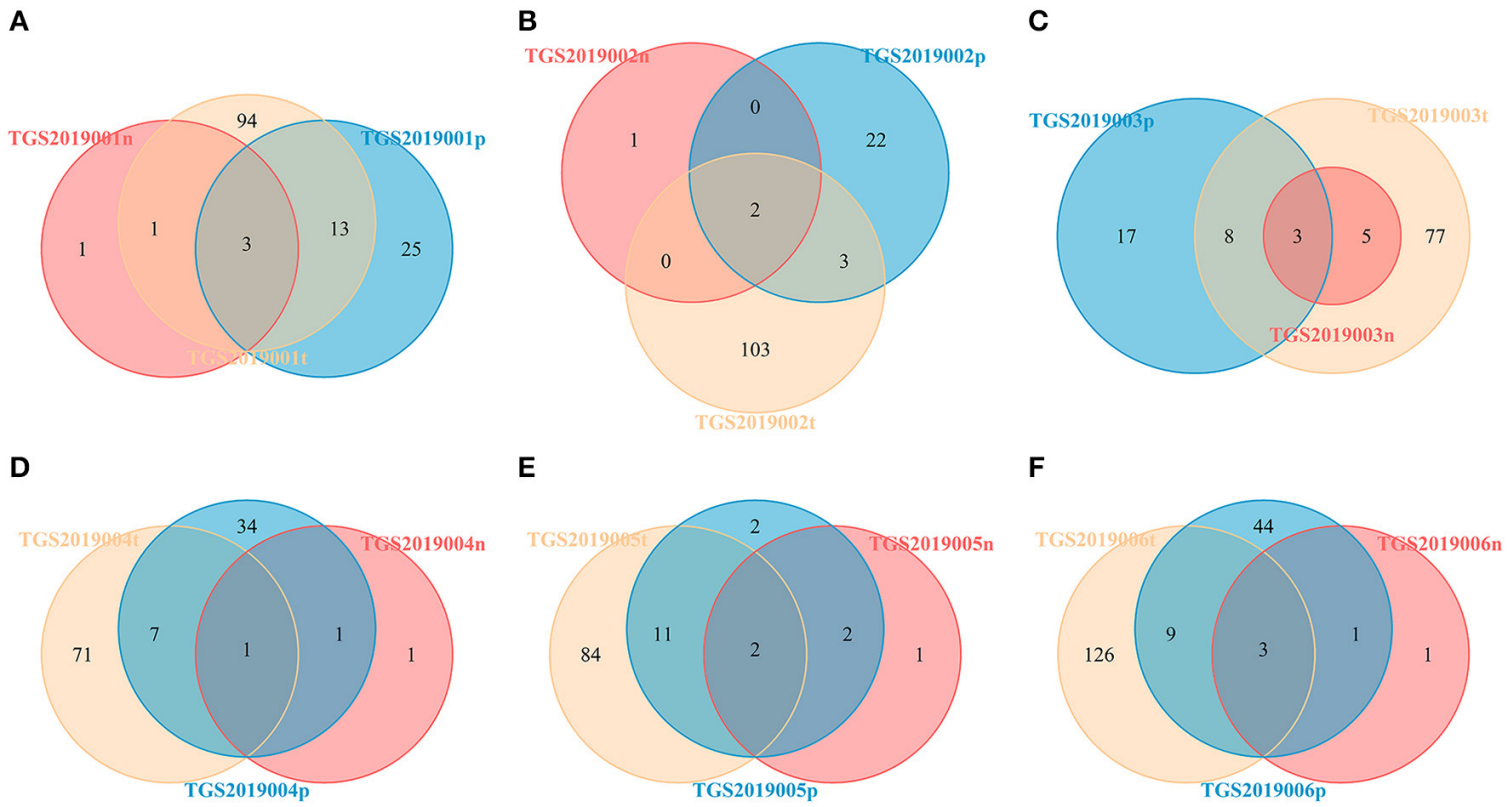
Venn diagram of V-J HECs of the PBMC, LN, and PTC (T) samples from six patients by IMonitor [TGS2019001 **(A)**, TGS2019002 **(B)**, TGS2019003 **(C)**, TGS2019004 **(D)**, TGS2019005 **(E)**, and TGS2019006 **(F)**]. The number of shared CDR3 V-J HECs between each patient's PBMC, LN, and PTC samples was 1–3. The number of unique V-J HECs in the PTC sample was greater than that in the PBMC sample, and both numbers were higher than that in the LN sample. “-t” indicates PTC tissues; “-n” indicates LNs; “-p” indicates PBMCs.

For each patient, the number of CDR3 V-J HECs at the amino acid level shared by the PBMC, LN, and PTC samples in each patient was 1–3. The number of unique V-J HECs in the PTC tissue was greater than that in the PBMCs, and both numbers were higher than that in the LNs (Figure 7). This finding was similar to CDR3 HECs at the amino acid level.

## Discussion

In this study, TCRβ CDR3 profiling was performed on PBMCs, LNs, and PTC tissue from six patients, and bioinformatics analysis was performed by IMonitor, MiXCR, and VDJtools. The results from IMonitor were generally similar to the results from MiXCR and VDJtools.

The unique CDR3 representing the T cell repertoire richness had significant differences among the three groups (*p* < 0.05), indicating that the CDR3 categories of LNs were higher than those of the PBMCs, and both values were higher than those of PTC tissues. Moreover, the CDR3 diversity index of the three groups, that is, the Shannon diversity index, D50, Inverse Simpson index_mean, and normalized Shannon's diversity index_mean were significantly different (*p* < 0.05). The diversity of the LNs was higher than that of the PBMCs and PTC tissues, and there was no significant difference between that of the PBMCs and the PTC tissues. The number of CDR3 HECs and CDR3 V-J HECs at the amino acid level in the PBMCs, LNs, and PTC tissues showed significant differences (*p* < 0.05). The number of CDR3 HECs and the rate of CDR3 V-J HECs in the PTC tissue was higher than that in the PBMCs, which was higher than that in the LNs. Although there was no significant difference in the total TCRs of the three tissue groups, the unique CDR3 sequences in the lymph node tissues were the highest and were relatively uniform, while the unique CDR3 sequences in the PBMC samples and PTC tissues were relatively few and relatively uneven, especially for the PTC tissues. This finding suggests that certain types of T cells may be generated and activated in PTC tissue and that antitumor immunity occurs directly.

Unique TCRs reflect the richness of TCRs in the sample and partially shows the diversity of TCRs. Some studies have compared the unique TCR CDR3s of cancer tissues with adjacent normal tissues and found that in certain malignant tumors, there were more unique TCR CDR3s in cancer tissues than in adjacent normal tissues, such as colon cancer (13) and breast cancer (14), and it was inferred that more T cells are recruited from the tumor tissue. However, opposite results, such as those in bladder cancer (15) and breast cancer (16), were also reported. The unique CDR3s of LNs were higher than those of peripheral blood and cancer tissues in this study.

TCR CDR3 diversity includes not only evaluating the number of unique CDR3 sequences in a sample but also evaluating the relative abundance of CDR3. Many studies have examined the diversity of TCR clones of different tissues (17, 18). In colon cancer research, the TCR diversity of cancer tissues was lower than that of adjacent tissues (13). The TCR diversity of cancer tissue was higher than that of normal lung tissue in a lung cancer study (19). The difference in TCR diversity between cancer tissues and adjacent tissues or blood samples among different tumor types might be due to the different tumor immune microenvironments. In this study, the TCR CDR3 HECs and the TCR CDR3 V-J HECs of different samples in the same group or from the same patient had very limited overlap. Some studies had similar conclusions. A study that focused on TCR sequencing of ovarian cancer and ascites showed that the TCR sequence of tumor infiltrating T cells and ascites T cells in tumor tissues overlap was very limited, even if the tumor tissue and ascites immune microenvironment in the same patient showed a major difference (18). Lu et al. studied the TCRβ sequencing of different cancer tissues in a patient with multifocal PTC and found that the TCRβ repertoire varied among seven cancer tissues from PTC (8). Their study also indicated that nonsynonymous somatic mutations could induce T cell proliferation and immune checkpoint inhibitors could be a promising therapy. Therefore, besides the intratumor heterogeneity, different T cell proliferation that due to different neoantigens or other Immunogenic antigens derived from somatic mutations may also contribute to the CDR3 diversity of a PTC tissue and may result in higher HEC rate but lower diversity when compared with non-tumor samples. Furthermore, the differences in the PTC tissues, PBMCs or LNs might be related to the inconsistency of HLA typing of each patient. Recognition of antigens by T cells requires the presentation of antigens by HLA. Different HLA types might present different antigens, and the corresponding TCRs for recognition could be different.

TCR diversity of cancer tissues or peripheral blood could also be used as an indicator of prognosis of patients with various cancers (20), such as breast cancer (21), liver cancer (22), nasopharyngeal carcinoma (23), diffuse large B-cell lymphoma (24), malignant melanoma (25), etc. Some studies have suggested that targeted immunotherapy, such as CTLA-4 inhibitors, could promote the reconstruction of TCRs and increase their diversity (26). Therefore, changes in the peripheral blood TCR repertoire may be used to monitor the body's response to immunotherapy. In many types of tumors, a higher degree of T cell infiltration, higher TCR diversity in peripheral blood (27), and lower diversity in tumor-infiltrating TCRs are all related to a better response to immunotherapy (28). High-frequency TCR clones may be T cells that are associated with antitumor immune responses (29). Maybe the CDR diversity in cancer tissue alone is insufficient to explain whether an immune response is more efficient against PTC or not. On the one hand, the relationship between TCR CDR3 diversity of cancer tissues, peripheral blood or LNs and prognosis or immunotherapy of PTC needs further study. On the other hand, antitumor immune responses are believed to occur and are more obvious in cancer tissues (PTC tissues) than in LNs and in peripheral blood (PBMCs) of PTC in this study, which might explain why PTC has a slow progression and good prognosis. And this needs to be confirmed by further experimental research.

Different gene rearrangements could have different lengths of CDR3 sequences. Studies have shown that a shorter CDR3 length is related to CD4^+^ T cells in thymus tissue (30). CDR3 length distribution may be related to disease. Sun et al. conducted an immune sequencing study on the cancer tissues and paracancerous tissues of 5 PTC patients (9). These researchers found that the length distribution of IGH CDR3 was significantly different between the cancer tissue group and the paracancerous tissue group, while the length of TCRβ CDR3 was not significantly different between the two groups. The length distribution of TCRβ CDR3 in this study was not significantly different among the PTC, PBMC and LN groups in this study, which indicated that the length of TCRβ CDR3 might not be a factor that distinguishes cancer tissues from other tissues in PTC.

The number of V-J recombinations is a key feature that accurately reflects the antigen recognition characteristics of the TCR CDR3. No V-J HEC was shared in any of the six samples within the three groups. Sun et al. found that TRBV2/TRBJ1-2 and TRBV2/TRBJ1-1 were the most common in the PTC group and the adjacent tissue group, and they are considered markers of the progression of PTC (9). V-J recombination as a marker of PTC requires further research.

Many studies have shown that in draining LNs, tumor antigens are presented to T cells by antigen presenting cells for the first time and cause tumor-specific T cell activation and proliferation (16). The degree of TCR overlap between the tumor tissue and the draining LN could be used to estimate the proportion of tumor infiltrating lymphocytes derived from the draining LN (31). Studies have shown that tumor tissue might also show T cell activation (32). In this study, compared with those of the LN tissue, the CDR3 diversity of the tumor tissue was lower, but the CDR3 HECs at the amino acid level were higher, the CDR3 V-J rearrangement at the amino acid level was lower, and the V-J HEC rate was higher, indicating that the activation and amplification of T cells may occur in the tumor microenvironment. In this study, the CDR3 HEC and CDR3 V-J rearrangement at the amino acid level of each patient overlapped more in tumor tissue and peripheral blood than in tumor tissue and lymph node tissue, indicating that activated T cells in tumor tissue might migrate to the blood. These T cells may be tumor immune markers and a tool for tumor immunotherapy.

## Conclusions

We observed differences in the TCR CDR3 characteristics among and within the PBMCs, PTC tissues and regional LNs of PTC patients, including the number of clonotypes, diversity estimation, CDR3 HECs at the amino acid level, CDR3 V-J HECs at the amino acid level. The results of the TCRβ repertoire of cancer tissues, peripheral blood and LN samples of PTC provide a reference for further study.

## Data Availability Statement

The datasets presented in this study can be found in online repositories. The names of the repository/repositories and accession number(s) can be found below: the NCBI BioProject, ID: PRJNA664708 (https://www.ncbi.nlm.nih.gov/bioproject/PRJNA664708).

## Ethics Statement

The studies involving human participants were reviewed and approved by Ethics Committee of Tianjin Medical University General Hospital. The patients/participants provided their written informed consent to participate in this study.

## Author Contributions

XH conceived the project. YW and YL analyzed the data and constructed the manuscript. YL and LC performed the experiments. RJ and KZ support the experimental techniques. LC, ZC, and XW collected clinical sample and information. All authors contributed to the article and approved the submitted version.

## Conflict of Interest

The authors declare that the research was conducted in the absence of any commercial or financial relationships that could be construed as a potential conflict of interest.

## Abbreviations

PTC: papillary thyroid carcinoma
PBMC: peripheral blood mononuclear cells
LN: regional lymph node
TCR: T cell receptors
TCRβ: T cell receptor beta-chain
CDR: complementary-determining regions
TILs: tumor infiltrating lymphocytes
HEC: high expansion clones.
